# SG-APSIC1099: Scoping review of cleaning of high-touch surfaces (HTSs) in inpatient wards

**DOI:** 10.1017/ash.2023.37

**Published:** 2023-03-16

**Authors:** Liang Hui Loo, Clara Chong Hui Ong, Sharifah Farhanah, Kyaw Zaw Linn, Xiaowei Huan, Kalisvar Marimuthu

**Affiliations:** National Centre for Infectious Diseases, Singapore; Infectious Disease Research and Training Office, National Centre for Infectious Diseases, Singapore; Infectious Disease Research and Training Office, National Centre for Infectious Diseases, Singapore; National Public Health & Epidemiology Unit, National Centre for Infectious Diseases, Singapore; Infectious Disease Research and Training Office, National Centre for Infectious Diseases, Singapore; Infectious Disease, Tan Tock Seng Hospital, Singapore

## Abstract

**Objectives:** High-touch surface (HTS) cleaning is critical to prevent healthcare-associated infections. However, HTS definitions and cleaning frequency vary across guidelines. We conducted a scoping review of published guidelines on HTS definitions and recommended cleaning frequency in inpatient wards. **Methods:** We searched national and societal guidelines on Google and PubMed using the following search terms: [(environmental cleaning/disinfection/housekeeping/sanitization), (hospital/healthcare/infection control prevention/inpatient/acute care), and (practice/guideline/guidance/methodology/protocol)]. We compared the guidelines’ HTS definitions, recommended cleaning frequency, and supporting evidence. **Results:** In total, 9 environmental cleaning guidelines were included: Centers for Disease Control and Prevention (CDC 2020); Asia Pacific Society of Infection Control (APSIC 2013); International Society for Infectious Diseases (ISID 2018); Joint Commission Resources (JCR 2018); National Health Service, United Kingdom (NHSUK 2021); Public Health Agency, Northern Ireland (PHANI 2016); Public Health Ontario, Canada (PHOC 2018); National Health and Medical Research Council, Australia (NHMRC 2019); Ministry of Health, Singapore (MOH 2013). These 6 guidelines identified 31 types of HTS: bed rails and frames [mentioned by 6 of 6 guidelines]; call bells, doorknobs and handles (5 of 6 guidelines); bedside tables and handles, light switches, overbed and tray tables, and sinks and faucet handles (4 of 6 guidelines); chairs and chair arms, edges of privacy curtains, IV infusion pumps and poles, keyboards, medical equipment, monitoring equipment, and telephones (3 of 6 guidelines); assist bars, counters, elevator buttons, toilet seats and flushes, transport equipment, and wall areas around the toilet (2 of 6 guidelines); and bedpan cleaners, beds, blankets, commodes/bedpans, dispensers, documents, mattresses, monitors, mouse, pillows, and touch screens (1 of 6 guidelines). The JCR, NHMRC, NHSUK guidelines did not define HTSs. The 6 guidelines recommended at least daily cleaning for HTSs, but ISID, JCR, and NHSUK guidelines did not mention HTS cleaning frequency. The CDC guidelines further specified at least once daily cleaning for inpatient wards and private toilets and twice daily for public or shared toilets. None of the guidelines cited any references for HTS cleaning frequency recommendations. **Conclusions:** There is no uniformity in HTS definitions among 6 guidelines, and the recommended HTS cleaning frequency in these guidelines was not supported by published evidence. Studies exploring optimal cleaning frequency of HTSs are needed.

